# Establishing the Socio-Economic Impact of Degenerative Cervical Myelopathy Is Fundamental to Improving Outcomes [AO Spine RECODE-DCM Research Priority Number 8]

**DOI:** 10.1177/21925682211039835

**Published:** 2022-02-17

**Authors:** Benjamin M. Davies, Richard Phillips, David Clarke, Julio C. Furlan, Andreas K. Demetriades, Jamie Milligan, Christopher D. Witiw, James S. Harrop, Bizhan Aarabi, Shekar N. Kurpad, James D. Guest, Jefferson R. Wilson, Brian K. Kwon, Alexander R. Vaccaro, Michael G. Fehlings, Vafa Rahimi-Movaghar, Mark R. N. Kotter

**Affiliations:** 1 Department of Neurosurgery, University of Cambridge, Cambridge, United Kingdom; 2 Myelopathy.org, International Charity for Degenerative Cervical Myelopathy, United Kingdom; 3 Goffin Consultancy, Canterbury, United Kingdom; 4 KITE Research Institute, University Health Network, Toronto, Ontario, Canada; 5 Division of Physical Medicine and Rehabilitation, Department of Medicine, University of Toronto, Toronto, Ontario, Canada; 6 Department of Clinical Neurosciences, Edinburgh, United Kingdom; 7 Department of Family Medicine, McMaster University, Hamilton, Ontario, Canada; 8 Division of Neurosurgery, Department of Surgery, University of Toronto, Toronto, Ontario, Canada; 9 Department of Neurological Surgery, Thomas Jefferson University, Philadelphia, PA, USA; 10 Department of Neurosurgery, University of Maryland School of Medicine, Baltimore, MD, USA; 11 Department of Neurosurgery, Medical College of Wisconsin, Wauwatosa, WI, USA; 12 Department of Neurosurgery and The Miami Project to Cure Paralysis, The Miller School of Medicine, University of Miami, Miami, FL, USA; 13 Department of Orthopedics, Vancouver Spine Surgery Institute, The University of British Columbia, Vancouver, British Columbia, Canada; 14 Department of Orthopaedic Surgery, Rothman Orthopaedic Institute, Thomas Jefferson University, Philadelphia, PA, USA; 15 Department of Neurosurgery, Sina Trauma and Surgery Research Center, Tehran University of Medical Sciences, Tehran, Iran

**Keywords:** cervical myelopathy, cervical spondylosis, cervical stenosis, disc herniation, ossification posterior longitudinal ligament, degeneration, research priorities, health economics, socioeconomics, policy

## Abstract

**Study Design:**

Literature Review (Narrative).

**Objective:**

To contextualize AO Spine RECODE-DCM research priority number 5: What is the socio-economic impact of DCM? (The financial impact of living with DCM to the individual, their supporters, and society as a whole).

**Methods:**

In this review, we introduce the methodology of health-economic investigation, including potential techniques and approaches. We summarize the current health-economic evidence within DCM, so far focused on surgical treatment. We also cover the first national estimate, in partnership with Myelopathy.org from the United Kingdom, of the cost of DCM to society. We then demonstrate the significance of this question to advancing care and outcomes in the field.

**Results:**

DCM is a common and often disabling condition, with a significant lack of recognition. While evidence demonstrates the cost-effectives of surgery, even among higher income countries, health inequalities exist. Further the prevalent residual disability in myelopathy, despite treatment affects both the individual and society as a whole. A report from the United Kingdom provides the first cost-estimate to their society; an annual cost of ∼£681.6 million per year, but this is likely a significant underestimate.

**Conclusion:**

A clear quantification of the impact of DCM is needed to raise the profile of a common and disabling condition. Current evidence suggests this is likely to be globally substantial.

## Introduction

Degenerative Cervical Myelopathy [DCM] is a neurological disorder arising from degenerative, arthritic, and/or congenital processes, causing cervical spinal cord dysfunction.^1,2^ DCM can result in a wide range of impairments and disabilities, including poor balance, limited mobility, loss of dexterity, sensory loss, bowel or bladder dysfunction, pain and in severe cases, paralysis.^1^ DCM is estimated to affect and contribute to neurologic dysfunction in up to 2% of the adult population.^3^ Given the increased prevalence of spinal degeneration with age, the incidence of DCM is expected to rise as populations age.^4^

Currently surgical decompression is the only evidence-based treatment recommended for progressive or moderate to severe disease.^5^ For most patients surgical intervention can halt disease progression and afford some meaningful recovery. However, recovery is normally incomplete, with some deficits and leaving individuals with life-long disabilities, dependency, unemployment, and mental health difficulties.^6,7^ In a comparison of SF-36 [the Short Form—(36) Health Survey of Quality of Life) scores of people with chronic disease, individuals with DCM were found to have the lowest quality of life scores.^7^ Moreover, the impact is not restricted to the individual, with a quality of life burden demonstrated among their family and/or acquaintance carers.^8^ Therefore, efforts to address and improve DCM outcomes should be a critical public health priority.

AO Spine RECODE-DCM (aospine.org/recode) [REsearch objectives and COmmon Data Elements for DCM] is an international consensus project which aims to accelerate knowledge discovery that can improve outcomes by developing a set of research tools.^9^ These include a James Lind Alliance research priority setting partnership, which brought together both individuals living and working with DCM to establish the most important unanswered questions. Research prioritization aims to catalyze progress by consolidating resources on key knowledge gaps.^10^ The Number 8 priority identified was to establish the socio-economic impact of DCM. The term socio-economic impact was used here to encompass the health-economic impact on the individual and society.

This article aims to contextualize: (a) the significance of this question; (b) to explain what is meant by socio-economic impact and how it can be measured; (c) to summarize the current evidence from within DCM and to provide a current best estimate, and (d) illustrate why this is a critical knowledge gap for the field that needs to be overcome to help improve outcomes.

### What Is Meant by Socio-Economic Impact, and How Can It Be Measured?

In this priority, the wording “socio-economic impact” was used to represent both the health-economic impact to the individual and to society. Health economics is the application of economic theory, decision-making models, and empirical techniques to analyze and make decisions on health and healthcare by taking into consideration the available resources as well as the values and needs from different stakeholders including individuals, health care providers, and governments.^11^ Simply stated, the aim of health technology assessment is to provide techniques to help manage limited resources most efficiently to achieve the best outcomes in populations.

There are several techniques that can be used largely depending on the perspective of the intended audience and the availability of data.^12^ When developing assessments, it is important to include the costs that are particularly relevant to the audience. For example, patients are most interested in the outcomes of the treatment and may have little or no interest in the cost of providing it (unless they are directly paying for it); the provider, however, wants satisfied patients but, more importantly, needs to be able to provide the treatment as cost-efficiently as possible, which means achieving the maximum benefit using the least resources (including money, time and manpower). Finally, the external payer (i.e. governments or private healthcare insurance) is looking for the most efficient means of providing a range of effective treatments within a limited budget. In summary, when undertaking health economic analysis, only those costs and/or benefits that are relevant to the particular audience or purpose should be included.

Taking each audience in turn, the primary data needs are:

Patient: clinical benefits, safety, and quality of life.

Provider: Incidence, cost of managing the condition: cost of surgery, resource usage (bed stay, outpatient, and other visits).

Payer: as with the provider but also additional direct costs such as absenteeism, lost production, disability benefits, and tax lost due to the condition.

**Figure 1.**
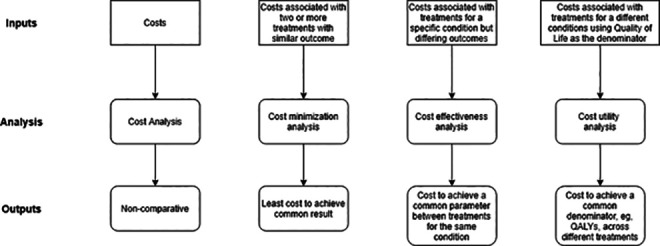
Summary of types of health economic analysis, including their principal purpose (represented as Output) and data requirements (represented as Input).

In terms of the “how to measure the socioeconomic impact,” this depends on the purpose of the analysis and, more importantly, how generalizable the results need to be (Figure 1). The commonly used methods include:

#### Cost analysis

This is a basic assessment of the costs of managing a condition without any consideration of the outcomes. The result cannot be used to compare with other treatments for the same condition, nor across treatments.

#### Cost minimization analysis

This is the simplest form of comparative analysis where the same outcome is possible using different treatments. In this context, the least costly treatment is deemed the most cost effective. For example, different antibiotics being used to treat a chest infection or, in the case of DCM, it might be a comparison of different surgical techniques where the same outcome will be achieved but at different costs of surgery.

#### Cost effectiveness analysis (CEA)

This is a method to compare both the costs and health outcomes of one or more interventions by estimating how much it costs to gain a health outcome unit like a life year gained or a death prevented.

#### Cost utility analysis (CUA)

The aim of a CUA is to attach a monetary value to outcomes to allow comparison across conditions. The most frequent common denominator is the Quality Adjusted Life Year (QALY), a generic measure of disease burden, which includes both the quality and quantity of life.

Quality of life can be assessed in a variety of ways such as:

**Time Trade Off** where an individual is asked to choose between remaining in a state of ill health for a period of time or being restored to perfect health but having a shorter life expectancy. The point where the respondent switch sides corresponds to the utility value for that health state.

**Standard gamble** where an individual is asked to choose between remaining in a state of ill health for a period of time or choosing a medical intervention that has a chance of either restoring them to perfect health or killing them. The point where the respondent changes opinion is considered the utility value for that health state.

**Visual Analogue Scale** where respondents are asked to rate a state of ill health on a scale from 0 to 100, with 0 representing being dead and 100 representing perfect health.

A generic scale which can be used in any condition that gives a weight associated with a particular health state is to use standard descriptive systems such as the EuroQol Group’s EQ-5D questionnaire, which categorizes health states according to 5 dimensions: mobility, self-care, usual activities (e.g., work, study, homework or leisure activities), pain/discomfort and anxiety/depression. While the SF-36 is the most commonly used quality of life score in DCM research today,^13,14^ for health economic analysis it must be mapped to the SF6D healthy utility.^15,16^

The result is a calculated index that ranges from 1 (perfect health) to 0 (dead); so one QALY equates to 1 year in perfect health. By associating the effect of treatment on quality of life and applying the cost of getting there, the cost/QALY can be estimated.

Several authorities use the Incremental Cost Effectiveness Ratio (ICER) to determine the value of new treatments. The ICER can be defined as:


$$\frac{\mathrm{cost of new treatment}-\mathrm{cost of standard treatment}}{\mathrm{QALY of new treatment}-\mathrm{QALY of standard treatment}}$$

For example, in England, the National Institute for Health and Care Excellence (NICE) uses a threshold of £20 000 to determine whether a new treatment should be introduced for use in the NHS.^17^ There are some exceptions to this threshold, for example, in certain cancers, rare diseases, or where patients have limited life expectancy. These thresholds are therefore influenced by a number of factors and are typically set per healthcare system:^18^ the World Health Organisation recommends using a threshold based on Gross Domestic Product to personalize recommendations.^19^

### What Is the Current Health-Economic Evidence Within DCM, and Why Must This Improve?

Within DCM, the health-economic evaluation has been restricted to evaluations of treatment cost, in particular surgery.^20-23^ Across the board, these studies strongly confirm the overall cost-effectiveness of surgery.^21^ Moreover, as a single up-front relatively high cost, with benefit likely extended well beyond the follow-up period, the study by Witiw et al (2016) using a Markov transitional model to estimate a life-time benefit is a high-quality example. This study, using a Canadian cohort of 171 patients, demonstrated cost-effectiveness, as per the World Health Organization criteria, in 94.7% of estimates.^20^ Further examples of health economic comparisons between surgical techniques,^24^ and surgery versus non-operative management have also been conducted^22^ but are more limited based on the quality of reference data.^18^

While robust health-economic evidence is necessary to support the adoption of clinical treatments, particularly within single payer healthcare systems, these evaluations do not serve to fully characterize the complete burden of illness; for example, the cost at a societal level or for the individual. These are likely more fundamental to driving system-wide changes, including healthcare policy, social care policy, and increased research investment, fundamental to future healthcare gains in DCM. Moreover, the cost to an individual is often an important determinant of quality of life, and mental well-being, for which the burden is well demonstrated, but the drivers are not.^7^

However, to calculate the cost of illness, several key pieces of evidence are required:

1. How many people are affected?
2. At what age does the condition develop?
3. What are the direct costs of managing the condition?
4. What are the indirect costs of managing the condition?
5. What are the costs to the individual, their caregivers, healthcare providers, payer, and society at large?

Unfortunately, most of these questions are poorly defined in DCM.

### What Is a Current Best Estimate Within DCM?

Myelopathy.org (Cambridge, United Kingdom) is the first, and so far, only, charity dedicated to DCM. Launched in 2018, it hosts a growing and international community of individuals living with DCM but also working with DCM.^25^ Fundamentally it aims to increase awareness and improve outcomes for those living with the condition. As part of these objectives, it has recently commissioned the first dedicated report on the burden of illness in DCM. The report made use of the best available data within the UK.

The prevalence of DCM was estimated based on International Classification of Disease (ICD-10) codes M47.12, M50.0, M99.31/.41/.51 from the National Health Service hospital episode statistics data, from inception (2013) to the end of 2019. This dataset provides overall event data for England (including wait time, and primary and secondary ICD codes), and some demographic data (age and gender). Extrapolating this across the UK population gave an estimated incidence of 7.44/100,00 (±0.32), in keeping with the literature.

Age of presentation was calculated by combining mean hospital waiting times from the hospital episode statistics data (73.6 days) with time to diagnosis data (assumed to be a surrogate for data of referral) from the literature; 2 retrospective cohort studies with average waits of 1.25 to 2.2 years.^6,26^ This was then subtracted from the average age at presentation (62.1 years overall, or 51.3 years for those of working age, defined as 18 to 65 years) to estimate the average age at which patients have sufficiently severe symptoms to seek medical intervention in the UK (i.e. 59.9 years overall or 49.1 years for those of working age).

Healthcare treatment costs were also extracted from the UK National Health Service Database for the aforementioned cases. For the most recent year queried (2018-2019), the total cost of care for DCM in England was estimated to be £38 871 534; £9216 weighted average per hospital admission.

Productivity is typically calculated up to the age of retirement as it is assumed that once a person reaches retirement age, they are no longer considered to be contributing members of society. This is not the case for many older people as they may provide voluntary work or non-paid family support such as looking after grandchildren so that their parents can go out to work, but it is more difficult to assess the value of this. Furthermore, several elderly individuals continue working beyond the age of 65 years old either for their choice or need due to financial burden. Based on the average age of these cases at presentation, and an average age of retirement of 65, this equated to a potential 15.1 years of affected productivity. In a previous survey conducted by Myelopathy.org^6^ of those under the age of 65 (N = 537), 41% were unable to work due to their disability, 28% were employed full time, 14% employed part-time, and 7% retired.

To estimate lost personal income in the UK, this data was further restricted to UK respondents 18 to 65 (N = 199), looking for work (45%). In the absence of linked income data, average weekly income for the UK population was taken from the UK Office for National Statistics (ONS) [£511/week working full time and £139.52/week working part-time (based on the average part time employment of 16 hrs per week)] and Office for Economic Co-Operation (OECD).^27,28^ Assuming an average individual works 48 weeks per year, this equates to a potential loss of income through unemployment of £18 663 (ONS data) or £25 524 (OECD data). Therefore, assuming a 3% inflation rate (a standard assumption for health technology assessments), and a loss of productivity of 15.1 years, the lifetime inflated loss of income could be £347 112 using ONS figures or £474 719 using OECD values. Considering disability benefits, and using the UK ‘Universal Credit’ allowance for a single person, aged over 25, with limited capacity to work, of £9021.72/year, this would equate to a £9641 (51%) or £16 503 (65%) reduction in personal income. Based on the average age of the UK population in 2019 of 81.2, disability benefits on average would be claimed for 16.5 years in those age >65 years and 30.5 years in those hospitalized with DCM at the average age of 51.1 years (13.9+16.5).

**Figure 2.**
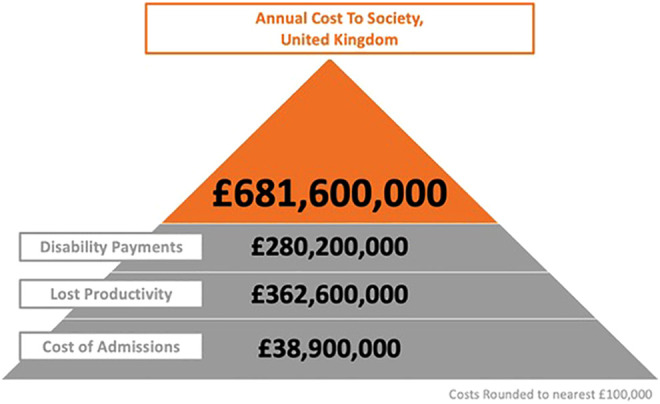
Estimate of overall annual cost to society, United Kingdom (myelopathy.org, United Kingdom). Total costs are round to nearest £100 000. In 2018, there were 4218 admissions, at an average cost of £9216 per admission: total cost of admission £38 900 000. Of these admissions, 2264 were within working age (defined as between 18 and 65), with an average 15.1 years remaining before retirement. Based on Pope et al^6^ up to 45% (1019) could be unable to return to work. The weighted annual average salary for 2018 is £25 524. For those of working age, lifetime loss of productivity is £362.6m. The weighted average disability payment (2020) is £9021.72, which based on 16.5 years of life remaining for those of >65 years and 30.5 years (13.9+16.5) in those hospitalized with DCM at the average age of 51.1 years, gives a total annual cost of £280.2m.

Based on these compiled best estimates, the following data can be integrated to yield an estimated (rounded to nearest £100 000) annual loss of productivity of £362.6m, disability benefits of £280.2m and therefore overall cost to society for this cohort of £681.6m (Figure 2).

### How Accurate Is This Estimate?

These calculations are based upon the best available data today. However, the calculations have their intrinsic limitations, including reliance on integrating aggregate data from different sources. While this, therefore, represents our best and only estimate so far, these data are likely to change. In terms of whether the estimate is under or over, there is much to suggest that these figures could increase substantially.

One of the challenges for population-level research in DCM is case ascertainment: today, there isn’t a specific ICD code for DCM.^29,30^ Instead, studies must select from various codes which can only approximate to varying degrees of specificity. The aforementioned report took a conservative approach to case identification using 4 codes, whereas many other studies have used additional codes, for example, in a population analysis for degenerative spinal conditions in Finland.^31^ For this reason, but also driven by recognized underdiagnosis, the true incidence, and prevalence of DCM is unknown. A recent meta-analysis of MRI cervical spine imaging in healthy cohorts identified a point prevalence of undiagnosed DCM of 2.4%.^3^ While this will likely include much milder disability (less likely to require surgery, more likely to be employed), this would undoubtedly increase the economic estimates for DCM on population numbers alone. AO Spine RECODE-DCM^9^ is seeking to establish consensus for an index term, to propose a unifying ICD code.

Furthermore, the hospital events data are unlikely to capture the complete cost of care; for example, data does not include primary care, while care within other specialties for subsequent disabilities such as pain, mental health, urology are not captured.

Traumatic spinal cord injury has more clearly defined the full economic impact of disease, benefiting from clear disease classification and within the UK, life-long follow up by Spinal Rehabilitation Services. This dataset has recently enabled an estimated lifetime cost of £1.43 billion based on current incidence rates.^32^ While the average disability, and economic impact on average will be higher per individual with traumatic spinal cord injury, given the high costs among even those with less disability (e.g. for individuals with ASIA Impairment Scale, Grade D, the lifetime cost per individual is estimated to be £0.47million) this would suggest the direct care costs for DCM are an underestimate.

Financial outcomes are almost never considered in DCM research to date,^13,14^ and while a significant impact on unemployment is logical, to date, this has been poorly characterized. As part of AO Spine RECODE-DCM,^9^ a core outcome set for DCM research is being developed. This includes some consideration of financial impact, which should better serve these analyses in the future.

### Are Health-Economic Models Fully Generalizable?

Health economic analysis within DCM has so far relied on aggregate data, and its extrapolation across subgroups may represent a further knowledge gap.^18^ Here we contextualize 3 important potential areas of Socio-Economic Status (SES), lower and middle vs. higher-income countries, and age, although there are likely more.

SES broadly refers to an individual’s economic and social circumstances and is typically assessed based on income, education, and/or occupation. The ONS (UK) uses occupation as an overall surrogate.^33^ SES has been linked to a wide range of health problems, driven by its interaction with key determinants of health: access to healthcare, environmental exposure, and health behavior.^34^

Within DCM, there are indicators of inequalities in care, albeit their impact is less certain. In a population study using the US National Inpatient Sample (2001-2010), private insurance status and white ethnicity were among independent predictors for receiving an anterior versus posterior surgical approach.^35^ Moreover, in their follow up study, the authors also identified that private insurance status was an independent predictor for receiving a multi-level (3+) instrumented fusion.^36^ Within the Myelopathy.org survey of people living with DCM, diagnostic delay was greater among those of black or African American ethnicity, with additional trends for those with lower educational qualifications.^6^ In a recent evaluation of patients undergoing ACDF (all indications) from a single US center over 8 years (N = 2387), state-funded (Medicare / Medicaid) patients had more co-morbidities, longer hospitalization, and more frequently returned for reassessment within 90 days than insurance funded patients.^37^ Taken together, this suggests that SES is likely an important determining factor of treatment costs and outcomes as well as a potential sources of unconsciousness bias during the decision making process for the individual’s treatment.

Of the health-economic data produced so far, surgery is considered cost-effective across age groups.^20-23^ However, age is recognized to impact DCM, associated with greater perioperative morbidity and a reduced, albeit still meaningful, amount of recovery.^38^ It is noteworthy that while the subject has received significant research attention, the majority of studied cohorts remain young (average ages 60 to 65), and it is not certain whether this data is generalizable to higher age groups, as increasingly seen in higher income countries.^39^ It is noteworthy that while a correlated surrogate, age is not necessarily the same as frailty, and this distinction may further need to be considered as this subgroup is addressed.^40^

The requirements in lower and middle income countries [LMIC] will also be different.^41^ Firstly, the different population demographics may have different epidemiology. Given the lack of robust health surveillance, and reduced access to diagnostic imaging, experiences from non-traumatic spinal cord injury (NTSCI) probably provide the only current estimates. Considering the few studies completed, the prevalence of degenerative spinal conditions remains high. For example, in a systematic review (2017) of NTSCI studies from sub-Saharan Africa, degenerative disorders accounted for 1.5-29% of cases. However, only 3 of the 19 studies included had access to an MRI scanner, with only 4-26% of patients receiving such a scan.^42^ A study using MRI in Ghana found 75.9% of NTSCI cases had degenerative disease of the spine.^43^ Secondly, management options may be influenced by the low-resource setting.^41^ For example, diagnostic imaging, such as MRI, may not be available or may be too costly for patients or their families to afford, and alternative diagnostic/treatment options sought. Clinical follow-up is often a challenge to provide, given the large distances patients may live from centers, and the poor communications infrastructure. Furthermore, surgical techniques may have to be adapted—for example, spinal implants are often not paid for by public health systems in LMIC, given their often-significant cost. Despite these challenges, providers are exploring alternatives; conventional myelography has been demonstrated as a potentially safe and effective alternative for selecting appropriate candidates for surgery in these settings^44^ while alternative, low cost, surgical implants are being sourced from manufacturers in India, China, and South Korea.^45^ However, these factors will contribute to a different health-economic model.

### Why Do We Need to Better Characterize the Socio-Economic Impact?

Despite its prevalence and clinical relevance, DCM remains under-recognized and under-treated. Increasing awareness has been identified as the number one priority by AO Spine RECODE-DCM [aospine.org/recode], fundamental to increasing diagnosis and timely treatment, but also much needed research investment. However, without a strong and robust health-economic argument for change, convincing healthcare leaders and funders to focus their attention on this public health priority will remain an uphill struggle. Notably these significant costs to the individual and society can be avoided, if DCM can be diagnosed and treated in a timely manner.

## Conclusions

The Socio-Economic impact of DCM is a critical knowledge gap. Indicators, including the current best estimate from Myelopathy.org, suggest the cost of illness is substantial. By properly determining and disseminating the information on the socio-economic impact of DCM, one may anticipate a change in the individual and societal value of investing in the care of patients with DCM and in the research and innovation for DCM.
